# Linker histone H1 and H3K56 acetylation are antagonistic regulators of nucleosome dynamics

**DOI:** 10.1038/ncomms10152

**Published:** 2015-12-09

**Authors:** Morgan Bernier, Yi Luo, Kingsley C. Nwokelo, Michelle Goodwin, Sarah J. Dreher, Pei Zhang, Mark R. Parthun, Yvonne Fondufe-Mittendorf, Jennifer J. Ottesen, Michael G. Poirier

**Affiliations:** 1 Department of Physics, The Ohio State University, Columbus, Ohio 43210, USA; 2 Biophysics Graduate Program, The Ohio State University, Columbus, Ohio 43210, USA; 3 The Ohio State Biochemistry Program, The Ohio State University, Columbus, Ohio 43210, USA; 4 Department of Biological Chemistry and Pharmacology, The Ohio State University, Columbus, Ohio 43210, USA; 5 Department of Molecular and Cellular Biochemistry, University of Kentucky, Lexington, Kentucky 40536, USA; 6 Department of Chemistry & Biochemistry, The Ohio State University, Columbus, Ohio 43210, USA

## Abstract

H1 linker histones are highly abundant proteins that compact nucleosomes and chromatin to regulate DNA accessibility and transcription. However, the mechanisms that target H1 regulation to specific regions of eukaryotic genomes are unknown. Here we report fluorescence measurements of human H1 regulation of nucleosome dynamics and transcription factor (TF) binding within nucleosomes. H1 does not block TF binding, instead it suppresses nucleosome unwrapping to reduce DNA accessibility within H1-bound nucleosomes. We then investigated H1 regulation by H3K56 and H3K122 acetylation, two transcriptional activating histone post translational modifications (PTMs). Only H3K56 acetylation, which increases nucleosome unwrapping, abolishes H1.0 reduction of TF binding. These findings show that nucleosomes remain dynamic, while H1 is bound and H1 dissociation is not required for TF binding within the nucleosome. Furthermore, our H3K56 acetylation measurements suggest that a single-histone PTM can define regions of the genome that are not regulated by H1.

## Abstract (web summary)

The linker histone H1 is highly abundant and regulates DNA accessibility by compacting chromatin. Here the authors analyze transcription factor binding to nucleosomes and show that histone H1 suppresses unwrapping but does not directly block the binding of transcription factors.

## Graphical abstract

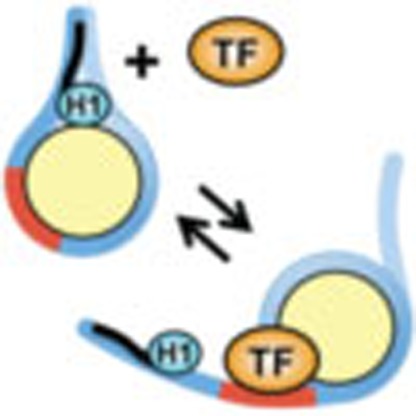

Genomic DNA in eukaryotes is repeatedly wrapped into nucleosomes to form chromatin^1^,^2^. The nucleosome contains ∼146 bp of DNA wrapped ∼1.65 times around a histone octamer with two copies each of histones H2A, H2B, H3 and H4 (ref. 3). These nucleosomes are further condensed via the linker histone, H1, an abundant eukaryotic protein. There is about one H1 protein per nucleosome in human somatic cells^4^, though H1:nucleosome ratios in other species vary significantly^5^,^6^. H1 binds linker DNA and the nucleosome to form a chromatosome^7^,^8^, which protects an additional 20 bp (168 bp total) from nuclease digestion^9^, thus shortening linker length and condensing chromatin. This organization of genomic DNA functions to regulate DNA accessibility to transcription and DNA repair machinery.

H1 contains three domains: the relatively short (∼30 amino acids) N-terminal domain, a long (∼100 amino acids) C-terminal domain, and a central winged helix globular domain (WHD)^10^. The WHD of H1 appears to bind to the dyad of nucleosomes^10^,^11^,^12^, while the exact binding of the other two domains remains largely unclear. The positively charged C-terminal tail is required for tight binding^13^, formation of folded chromatin^14^,^15^ and interacts with the negatively charged linker DNA^16^.

Nucleosome and chromatin compaction regulate TF accessibility to their target sites in promoters^17^,^18^. This regulation was visually confirmed by a recent super-resolution microscopy study that showed chromatin is decondensed at transcription sites^19^. Ultraviolet and chemical cross-linking studies of H1 indicate that it is depleted in actively transcribed regions^14^,^20^,^21^, suggesting that H1 depletion is required for transcriptional regulation. However, a separate cross-linking study that focused on the tail domains of H1 found that cross-linking levels were the same for active and inactive regions, indicating that H1 remains associated to the DNA via its C-terminal tail during transcription^22^. *In vitro* studies have shown that H1 represses TF binding to DNA sites within nucleosomes^18^,^23^ and that hyperacetylated histones reduce H1 repression of TF binding^23^. However, the mechanisms by which H1 represses DNA accessibility to TF binding, which histone acetylation sites impact H1 function and how acetylation functions to regulate H1 remain undetermined.

In humans, there are 11 H1 isoforms. Four are only expressed in germ line cells, while seven are expressed in somatic cells^24^. Of the somatic H1 isoforms, H1.1, H1.2, H1.3, H1.4 and H1.5 are expressed during replication, while H1.*x* and H1.0 are expressed throughout the cell cycle. The WHD is well-conserved between each isoform, while the N and C termini diverge significantly. Variation in amino-acid sequence is reported to impact affinity to chromatin *in vitro*^25^ and exchange rate *in vivo*^26^. The C-terminal charge of H1 isoforms correlates with relative exchange rates *in vivo*. However, differences in histone isoform affinities to chromatin *in vitro* were not consistent with exchange rates *in vivo*. This suggests that other factors could differentially impact H1 isoform function, but these functional differences are not currently well-understood.

Here we report Förster resonance energy transfer (FRET) and protein-induced fluorescence enhancement (PIFE) studies of the human linker histone isoforms H1.0, H1.*x* and H1.2 and TF binding within nucleosomes. We find that H1.0 suppresses by threefold TF binding to a recognition sequence located within the DNA entry–exit region of the nucleosome. TF binding occurs while H1.0 remains bound to linker DNA, indicating that H1.0 dissociation is not required for TF binding within the nucleosome. Instead it appears that nucleosomes continue to partially unwrap and rewrap as they do without H1.0 bound^27^,^28^,^29^ but with a threefold lower probability of unwrapping. To confirm that this is not isoform specific, we also investigated the influence of histone isoforms H1.*x* and H1.2 on TF binding. We find that they function similarly to H1.0 in that they also suppress TF binding but remain bound to linker DNA as a TF binds within the nucleosome. We then investigated the impact of two histone PTMs: H3K56ac, which is located near the DNA entry–exit region of the nucleosome and increases nucleosome unwrapping^30^,^31^,^32^, and H3K122ac, which is located near the nucleosome dyad symmetry axis and reduces binding of the H3–H4 tetramer to DNA^33^. Both of these modifications occur within actively transcribed genes^34^,^35^,^36^. H3K56ac abolishes H1 repression of TF binding, while H3K122ac did not influence H1 function. H3K56ac does not regulate H1 by altering its binding to linker DNA. Instead, it appears that increased nucleosome unwrapping by H3K56ac antagonizes H1 suppression of unwrapping and suggests that H3K56ac can define regions of the genome where DNA accessibility is unaffected by H1.

## Results

### Fluorescence measurements of H1.0 binding to nucleosomes

**Figure 1:**
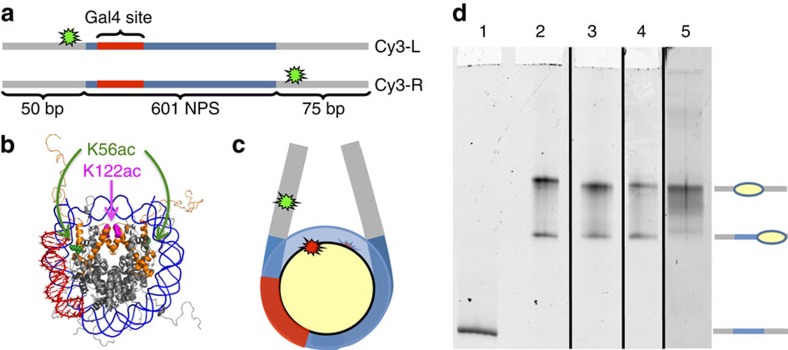
Design and characterization of nucleosomes used for FRET and PIFE experiments. (**a**) Schematic of Cy3 labelled 272-bp DNA construct containing Widom 601 nucleosome positioning sequence^42^ (blue) with the Gal4-2C target sequence (red) used in both the FRET and PIFE experiments. (**b**) X-ray crystal structure of nucleosome (PMID: 1KX5) showing the location of the PTMs H3K56ac (green) and H3K122ac (magenta). (**c**) Cartoon depiction of nucleosome constructs showing location of fluorophores. The Cy5 fluorophore is on H2A at K119C. (**d**) 5% Native PAGE showing nucleosomes. Lane 1: DNA, Lane 2: Unmodified nucleosomes, Lane 3: H3K56Q, Lane 4: H3K56ac, Lane 5: H3K122ac. The top band is nucleosomes on the positioning sequence, the bottom band are nucleosomes at the end.

**Figure 2:**
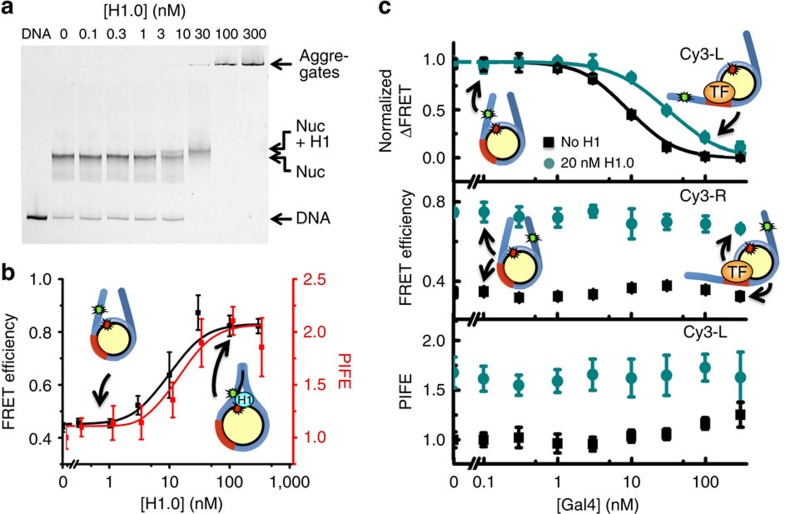
Characterization of fluorescence system for observing H1 binding to nucleosomes. (**a**) EMSA showing H1.0 binding to nucleosomes. Nucleosome band begins to shift at 10 nM H1.0, and is completely shifted at 30 nM H1.0. In addition, a small fraction of nucleosomes do not migrate into the gel at 30 nM H1.0, which suggests the nucleosomes are all bound with H1.0 and on the verge of aggregating, The nucleosomes do not migrate into the gel at 100 and 300 nM, which indicates they are aggregating at these H1.0 concentrations. (**b**) FRET efficiency versus H1.0 concentration (black) measured with Cy3–Cy5 labelled nucleosomes and PIFE signal versus H1.0 concentration (red) measured with Cy3 only nucleosomes. As H1.0 binds, it wraps nucleosomes, thus increasing FRET efficiency. In addition, as H1.0 binds, it interacts with the Cy3 on the DNA increasing PIFE. (**c**) Top: Normalized change in FRET efficiency: (FRET−FRET_final_)/(FRET_initial_−FRET_final_) of Cy3-L nucleosomes versus Gal4 concentration with 0 nM and 20 nM H1.0. The data were fit to a Hill Curve. In the presence of H1, the *S*_1/2_ shifts indicating H1.0 inhibition of Gal4 binding within nucleosomes. Middle: FRET Efficiency of Cy3-R nucleosomes versus Gal4 concentration with 0 nM and 20 nM H1.0. The FRET efficiency is increased in the presence of H1.0, but does not change as Gal4 binds indicating that H1 is regulating unwrapping rather than sliding of the nucleosomes. Bottom: PIFE versus Gal4 concentration with Cy3 only nucleosomes. PIFE increases in the presence of H1.0, but does not change as Gal4 binds indicating that H1.0 remains bound to the linker DNA as it unwraps from the nucleosome. The error bars were determined from the s.d. of three independent measurements.

We developed two fluorescence systems to investigate H1 binding to nucleosomes in equilibrium. We decided to focus on H1.0 in initial studies since it is the most extensively characterized isoform^24^. The first fluorescence system takes advantage of the observation that H1.0 binding increases DNA wrapping into the nucleosome^7^. We prepared Cy3–Cy5 nucleosomes with Cy3-L DNA that has Cy3 attached to the eighth bp before the nucleosome positioning sequence and with Cy5 attached to K119C on H2A (Fig. 1a–c). These fluorophore positions result in a FRET efficiency of about 0.5 (Fig. 2b). To confirm that H1.0 binds these Cy3–Cy5 labelled nucleosomes, we used an electrophoretic mobility shift assay (EMSA) of H1.0 titrations (Fig. 2a). The nucleosome band begins to shift up into a chromatosome band at around 10 nM, which is consistent with previous studies of H1.0 binding^37^. At 30 nM we observe a complete shift to the chromatosome band, while above 30 nM the nucleosomes aggregate. We also assessed H1.0 binding to unlabelled nucleosomes (Supplementary Fig. 1) and determined that the fluorophores do not alter H1.0 binding to nucleosomes. We then used our first fluorescence system to determine the influence of H1.0 on the FRET efficiency, as a measure of DNA wrapping. As H1.0 is titrated, we observe an increase in the FRET efficiency to about 0.9 (Fig. 2b, Supplementary Fig. 2a), which is consistent with the observations that H1 increases nucleosome wrapping. The FRET measurements of H1.0 binding (excluding data points for aggregated nucleosomes) were fit to a Hill curve with a *S*_1/2_=9±6 nM, which is in agreement with our EMSA measurements and demonstrates that H1.0 binding can be detected by FRET measurements of DNA wrapping within the nucleosome.

The C-terminal tail of H2A interacts with H1.0 and is required for efficient H1.0 binding^38^. Because Cy5 is attached near the C terminus of H2A, we investigated whether the H1.0-induced FRET change is a result of increased wrapping or movement of the H2A C-terminal tail on H1.0 binding. We prepared Cy3–Cy5 nucleosomes with the Cy5 attached to H3 at V35C and conducted H1.0 titration experiments with these nucleosomes (Supplementary Fig. 3). We observe nearly the same binding isotherm for both Cy5 positions confirming that we are detecting H1.0 binding by changes in nucleosome wrapping.

The Cy3 peak in the fluorescence spectra of the H1.0 titrations with Cy3–Cy5 labelled nucleosomes does not significantly decrease as the acceptor peak increases as is expected during FRET (Supplementary Fig. 2a). This indicates that H1.0 binds in close proximity to the Cy3 label on the DNA, resulting in PIFE. We took advantage of this enhancement to directly detect H1.0 binding to the linker DNA of the nucleosome. We prepared nucleosomes with Cy3-L DNA, but without a Cy5 fluorophore. We carried out H1.0 titrations and determined the Cy3 fluorescence with H1.0 relative to the Cy3 fluorescence without H1.0. We find H1.0 induces a twofold increase in Cy3 fluorescence, which is a typical change in Cy3 fluorescence due to PIFE^39^,^40^. These PIFE measurements of H1.0 binding (excluding data points for aggregated nucleosomes) were fit to a Hill curve with a *S*_1/2_=13±4 nM, which is in agreement with our FRET and EMSA measurements (Fig. 2b). Uncertainties are determined from a weighted least-squares fit. This PIFE measurement provides an alternative measurement of H1.0 binding that is independent of the amount of DNA wrapped into the nucleosome.

### H1.0 suppresses nucleosome partial unwrapping

To investigate the mechanism by which H1.0 regulates TF binding within the nucleosome, we included a Gal4-binding site at the 8th through 26th base pairs of the nucleosome (Fig. 1a). Gal4 can bind to its target site when the nucleosome partially unwraps and transiently exposes the site^41^. At sufficient Gal4 concentrations, Gal4 traps the nucleosome in a partially unwrapped state, which can be detected by a reduction in FRET from nucleosomes containing the Cy3-L DNA (Fig. 2c). By measuring the change in FRET efficiency as a function of Gal4 concentration, we determine the *S*_1/2_ of Gal4 binding to partially unwrapped nucleosomes. The *S*_1/2_ is the concentration of Gal4 at which half of the nucleosomes are bound and is inversely proportional to the change in the probability that Gal4 is bound to its site. Gal4 occupancy is due to a dynamic equilibrium where Gal4 continually binds to a partially unwrapped nucleosome, transiently trapping it in this partially unwrapped state, and then dissociates allowing the nucleosome to rewrap^41^.

To determine the influence of H1.0 on Gal4 binding, we carried out Gal4 titrations with the Cy3–Cy5 nucleosomes containing Cy3-L DNA with and without 20 nM H1.0 (Fig. 2c). The changes in FRET efficiency were normalized and fit to a Hill curve. The *S*_1/2_ for Gal4 binding increased from 9.2±0.3 nM to 31±2 nM as the H1.0 concentration was increased from 0 to 20 nM, which implies a decrease in TF occupancy by a factor of 3.4±0.2 (Table 1). An H1.0 concentration of 20 nM was chosen as it is near the point of H1.0 saturation and does not induce nucleosome aggregation. We find that the Gal4-induced change in FRET efficiency at 20 nM H1.0 is similar to the FRET change without H1.0 (Supplementary Fig. 4), which indicates that Gal4 binds within nucleosomes in the presence of nearly saturating concentrations of H1.0. This suggests that H1.0 suppresses but does not block TF binding and is consistent with previous work by Workman and coworkers where they used EMSA studies to observe H1 regulation of TF binding within the nucleosome^18^.

To investigate whether H1 decreases DNA unwrapping of the nucleosome to reduce the probability for Gal4 binding within the nucleosome, we prepared nucleosomes with Cy3-R DNA (Fig. 1a). In this nucleosome construct the Cy3 fluorophore and the Gal4-binding site are on opposite sides of the nucleosome, so changes in FRET due to Gal4 binding to its target site can only be due to changes in nucleosome sliding but not nucleosome unwrapping. We carried out Gal4 titrations and find that Gal4 does not reduce the FRET efficiency with or without 20 nM H1.0 (Fig. 2c), which rules out nucleosome sliding. Therefore, our results indicate that even in the presence of H1.0, Gal4 binds within the nucleosome via DNA unwrapping, and that H1.0 regulates TF binding by suppressing the probability the nucleosome is partially unwrapped. Interestingly, in the absence of Gal4 and H1.0, the absolute FRET efficiencies of nucleosomes with Cy3-R are less than those with Cy3-L (Fig. 2b,c). This is consistent with previous reports of asymmetric nucleosome unwrapping fluctuations^42^,^43^,^44^.

### TF binding does not require H1.0 dissociation

There are two mechanisms for H1.0 repression of Gal4 binding: (i) a blocking mechanism and (ii) a reduced site exposure mechanism. H1.0 could function by keeping the nucleosome in a fully wrapped state and block TF binding when H1.0 is bound to the nucleosome. Gal4 would then bind to the nucleosome only when H1.0 dissociates and the nucleosome can partially unwrap. Alternatively, H1.0 could function to reduce DNA unwrapping while it remains bound. Gal4 would then bind to partially unwrapped nucleosomes with H1.0 bound. Here H1.0 shifts the unwrapping equilibrium but does not dissociate for a TF to bind.

To differentiate between these mechanisms, we investigated whether H1.0 dissociation is required for Gal4 to bind to partially unwrapped nucleosomes. We used Cy3 only labelled nucleosomes that contained Cy3-L DNA with the Gal4 target sequence (Fig. 1a). This construct detects H1.0 binding near the eighth base pair of the DNA outside of the nucleosome with PIFE (Fig. 2b). We carried out Gal4 titrations with nucleosomes containing Cy3-L and determined PIFE as a function of Gal4 concentration (Fig. 2c). We find that PIFE is not increased by Gal4 binding to nucleosomes (Fig. 2c, black squares), which demonstrates that the PIFE measurement of H1.0 binding is not influenced by DNA unwrapping. We then carried out Gal4 titrations in the presence of 20 nM H1.0. As expected from our H1.0 titration (Fig. 2b), H1.0 causes the PIFE to increase to a value of about 2. However, PIFE does not change as the Gal4 concentration is increased over the range where Gal4 binds within the nucleosome (Fig. 2c). This result indicates that H1.0 remains bound to the linker DNA as Gal4 binds to partially unwrapped nucleosomes and that interactions between H1.0 and linker DNA are important for this H1.0 function.

**Figure 3:**
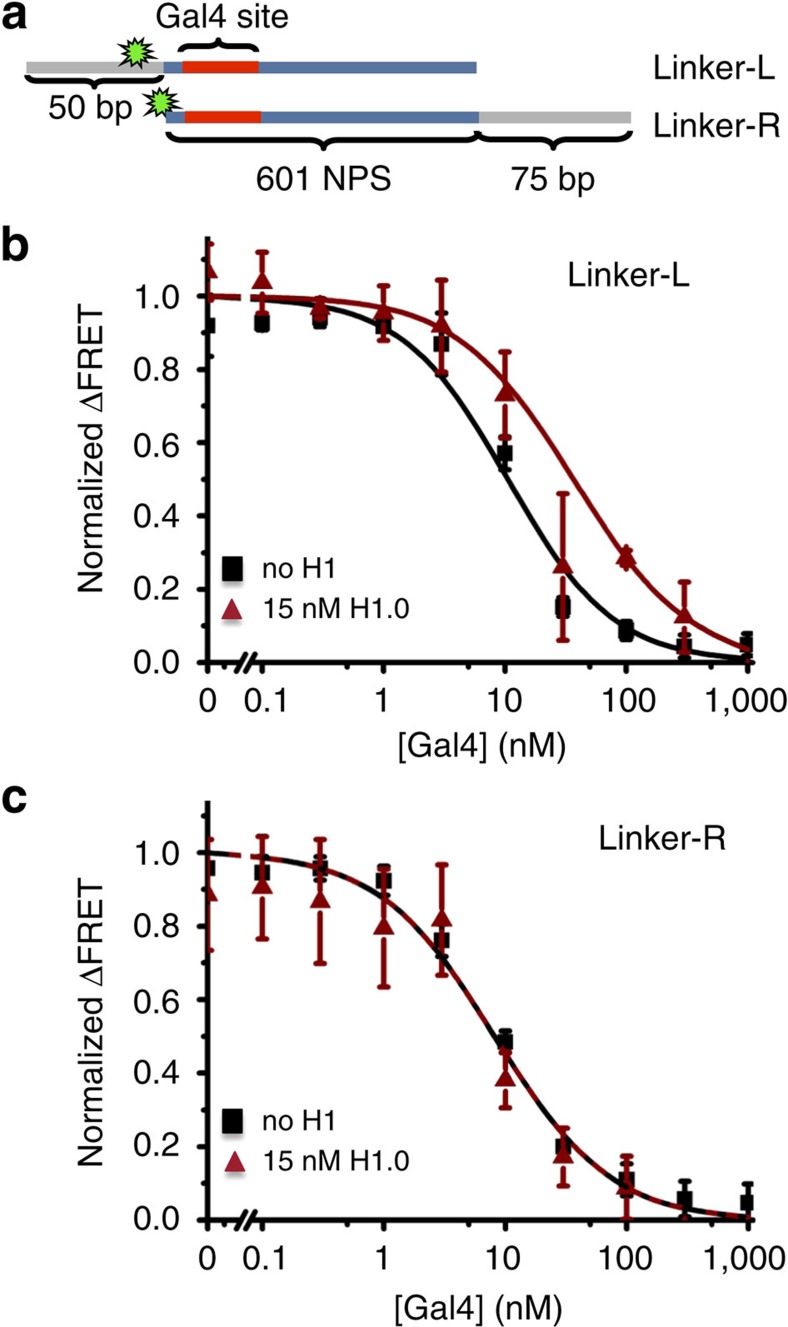
H1 effect on Gal4 binding to asymmetric nucleosomes. (**a**) DNA constructs used to make nucleosomes with DNA only on one side. (**b**) FRET efficiency as a function of Gal4 concentration with and without H1.0 with Linker-L nucleosomes in which the linker is on the same side as the Gal4 recognition sequence. (**c**) FRET efficiency as a function of Gal4 concentration with and without H1.0 with Linker-R nucleosomes in which the linker is on the opposite side of the Gal4 recognition sequence. These results show that linker DNA adjacent to the Gal4-binding site is required for H1.0 suppression of Gal4 binding. The error bars were determined from the s.d. of three independent measurements.

This observation suggested to us that only the linker DNA on the side of the nucleosome with the Gal4-binding site is required for H1.0 repression of Gal4 binding. To investigate this we prepared Cy3–Cy5 labelled nucleosomes with linker DNA on only one side of the nucleosome. Nucleosomes containing Linker-L DNA have linker DNA only on the same side of the nucleosome as the Gal4-binding site, while nucleosomes reconstituted with Linker-R have linker DNA only on the side of the nucleosome opposite to the Gal4-binding site (Fig. 3a). We first carried out H1.0 titrations with these nucleosomes and found that Linker-L nucleosomes have a similar FRET increase to Cy3-L nucleosomes (Supplementary Fig. 5), while the Linker-R nucleosomes have a significantly smaller change in FRET on H1.0 binding. This suggests that the full increase in nucleosome wrapping by H1.0 on one side of the nucleosome requires linker DNA on the same side of the nucleosome. We then carried out Gal4 titrations with and without 15 nM H1.0 and determined the change in the Gal4 *S*_1/2_ (Fig. 3b,c). We used 15 nM H1.0 because the Linker-L nucleosomes had a more abrupt change in FRET for increasing concentrations of H1.0 and we wanted to be sure there was no H1.0-induced aggregation. We find that H1.0 increases the Gal4 *S*_1/2_ with nucleosomes containing Linker-L DNA, while H1.0 does not increase the Gal4 *S*_1/2_ with nucleosomes containing Linker-R DNA (Fig. 3b,c). These results show that the linker DNA adjacent to the Gal4-binding site is required for H1.0 suppression of Gal4 binding.

Previous studies have shown that H1.0 binds DNA with only a slight lower affinity to binding nucleosomes^45^,^46^. This observation suggests the H1.0 binding to linker DNA significantly contributes to H1.0 binding to nucleosomes and is consistent with our observation that linker DNA adjacent to the Gal4-binding site is required for H1.0 suppression of Gal4 binding. Altogether, these results demonstrate that H1.0 functions by mechanism (ii) where it suppresses nucleosome unwrapping fluctuations while remaining bound to the linker DNA just outside of the nucleosome, which represses protein binding to sites within the nucleosome.

It is important to note that since we detect binding within the linker DNA with one Cy3 fluorophore eight base pairs from the NPS, additional H1 molecules could bind the nucleosome and not induce a change in PIFE. So, it is difficult for us to completely rule out the possibility that an additional H1 molecule binds the nucleosome. However, recent NMR^12^ and cryoEM studies^47^ suggest that one H1 molecule binds per nucleosome. Furthermore, our gel shift data suggest that there is only one H1.0 bound species before the nucleosomes aggregate, since we observe only one hypershifted nucleosome band. Since our PIFE measurements change nearly identically to the gel shift and FRET measurements, even if more than one H1.0 binds a single nucleosome, then the H1.0 that is responsible for the increase in FRET and the decrease in nucleosome unwrapping remains bound, while the nucleosome partially unwraps for Gal4 binding.

### H1 isoforms function similarly to suppress TF binding

**Figure 4:**
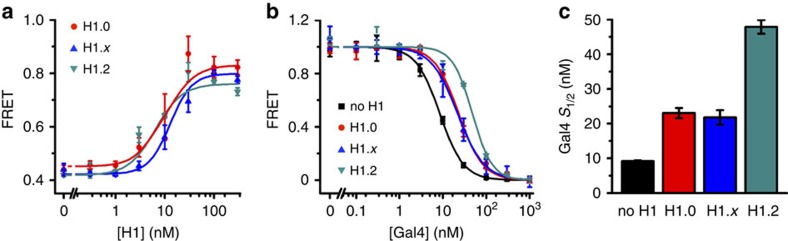
H1.2 and H1.*x* suppress Gal4 occupancy similarly to H1.0. (**a**) FRET efficiency measurements of H1 isoform titrations with Cy3–Cy5 labelled unmodified nucleosomes. H1.0 (*S*_1/2_=9±6 nM), H1.*x* (13±4 nM) and H1.2 (6±2 nM) binding causes the nucleosomes to wrap more, which increases the FRET efficiency. This allows us to measure H1 binding. (**b**) Normalized change in FRET efficiency of Cy3–Cy5 labelled unmodified nucleosomes for increasing Gal4 concentrations in the presence and absence of H1 isoforms. For titrations in the presence of H1 isoforms, the H1 concentration was set equal to the measured *S*_1/2_ of H1 binding to nucleosomes as measured in (**a**). An inhibition of Gal4 binding to the nucleosome was observed with all H1 isoforms. (**c**) Bar graph showing *S*_1/2_ values of Gal4 binding to the nucleosomes in the presence and absence of H1 isoforms measured by fitting the data in (**b**) to a Hill curve. The error bars in **a** and **b** were determined from the s.d. of three independent measurements. The error bars in **c** represents uncertainties from the weighted least squares fit.

There are seven different linker histone isoforms that are expressed in human somatic cells. We hypothesized that linker histone isoforms vary in their regulation of TF occupancy. To investigate this, we expressed and purified one replication dependent linker histone H1.2 and the other replication independent isoform H1.*x*. We carried out H1.2 and H1.*x* titrations with Cy3–Cy5 and Cy3–only labelled nucleosomes containing Cy3-L DNA and determined the changes in FRET (Fig. 4a) and PIFE (Supplementary Fig. 6). We find that H1.2 and H1.*x* induce similar changes in FRET and PIFE, and have a similar *S*_1/2_ to that of H1.0 (Table 2). This shows that both our FRET and PIFE measurements can be used to detect H1.2 and H1.*x* binding and that in our measurements they bind to nucleosomes with similar affinities. This result is in contrast to a previous study of post-translationally modified H1 isoforms isolated from rat brain, which reported that H1.0 binds nucleosome with a significantly higher affinity than H1.2 (ref. 25). Combined, these results suggest that differences in binding affinities could be due to differences in post translational modifications, rather than inherent differences between H1 isoforms.

We then used the FRET system to monitor Gal4 binding to nucleosomes containing the Cy3-L DNA in the presence of H1.2 and H1.*x*. To control for small variations in the linker histone binding affinity, we carried out the Gal4 titrations with H1.2, H1.*x* and H1.0 concentrations equal to their measured *S*_1/2_ (Fig. 4b). We found that H1.*x* suppressed Gal4 binding nearly identically to H1.0, while H1.2 suppressed Gal4 by twofold more than H1.0 or H1.*x* (Fig. 4c, Table 2). This indicates that while these linker histone isoforms suppress TF binding similarly, there are quantitative differences between isoforms that could allow them to differentially regulate DNA accessibility.

To investigate whether H1.2 and H1.*x* remained bound to linker DNA as Gal4 binds within the nucleosome, we carried out Gal4 titrations with Cy3 only labelled nucleosomes containing Cy3-L DNA. We find that the PIFE is not reduced as the Gal4 concentration is increased to the regimes where our FRET measurements indicate it is bound (Supplementary Fig. 7). This indicates that alternate linker histone isoforms function similarly to H1.0 and remain bound to linker DNA as the nucleosome unwraps and proteins such as TFs bind to partially unwrapped nucleosomes. This mechanism therefore appears to be isoform independent.

### H3K56ac abolishes H1.0 inhibition of Gal4 binding

**Figure 5:**
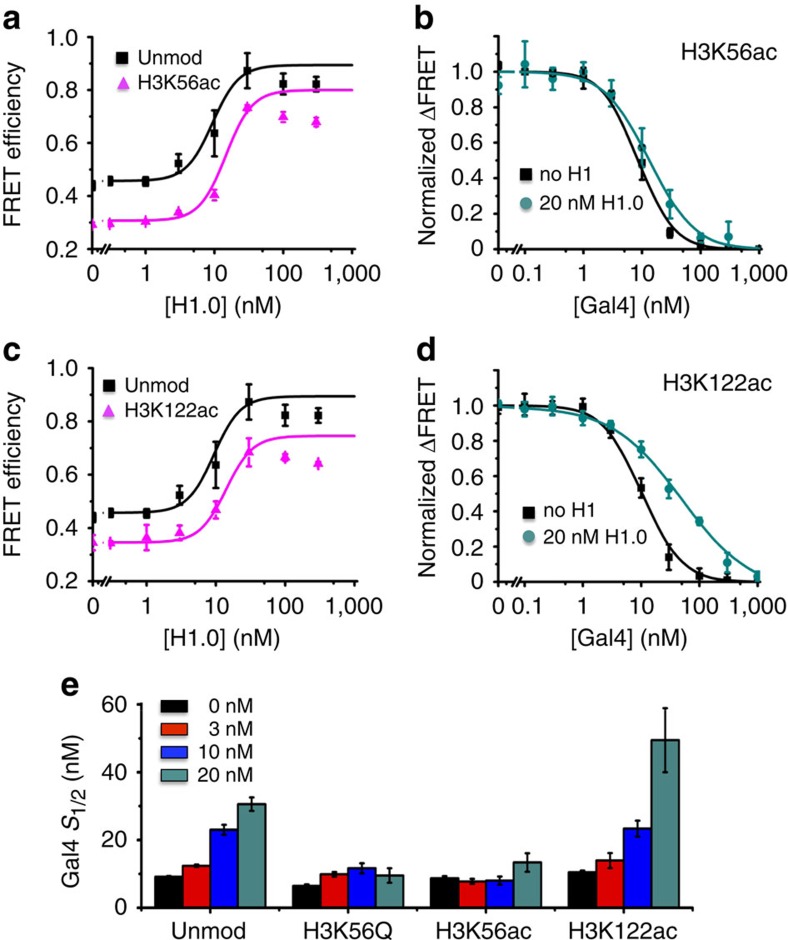
H1 inhibition of Gal4 binding with modified nucleosomes H3K56ac and H3K122ac (**a**) FRET efficiency as a function of H1 concentration for unmodified and H3K56ac nucleosomes. Data were globally fit to a Hill curve showing a Hill coefficient of 2.3±0.9 (**b**) FRET efficiency of Gal4 titration with H3K56ac 0 nM and 20 nM H1. Data were fit to a Hill curve with no shift in the *S*_1/2_. (**c**) FRET efficiency of H1 titration with unmodified and H3K122ac nucleosomes. Data were included in the global fit used in A. (**d**) FRET efficiency of Gal4 titration with H3K122ac nucleosomes with 0 and 20 nM H1. Data were fit to a Hill curve. The shift in the *S*_1/2_ is similar to that of unmodified nucleosomes. (**e**) Bar graph of the *S*_1/2_ from Gal4 titrations with increasing concentrations of H1 for unmodified, H3K56Q, H3K56ac and H3K122ac nucleosomes. The error bars were determined from the s.d. of three independent measurements.

The histone PTM, H3K56ac, occurs within gene promoters, correlates with enhanced transcription factor binding^48^,^49^ and is involved in regulating transcription^34^,^35^. We and others have previously reported that H3K56ac enhances site accessibility by increasing nucleosome unwrapping^31^,^32^,^50^. Therefore, we hypothesized that H3K56ac could influence linker histone repression of DNA accessibility. To investigate the impact of H3 acetylation on H1 binding and its ability to inhibit Gal4 binding, we prepared Cy3–Cy5 labelled nucleosomes with histone octamer containing H3K56ac and Cy3-L DNA. We carried out H1.0 titrations with H3K56ac nucleosomes and determined there was no significant change in the H1.0-nucleosome binding as assessed by FRET-detected changes in nucleosome wrapping in the entry–exit region (Fig. 5a, Table 1). We then performed Gal4 titrations with these nucleosomes at various H1.0 concentrations (Fig. 5b,e, Table 1). In contrast to unmodified nucleosomes discussed in the previous section, the Gal4 titrations with H3K56ac nucleosomes showed essentially no shift in the Gal4 *S*_1/2_ at increasing H1.0 concentrations, indicating that H3K56ac abolishes H1.0 regulation of TF binding.

H3K56Q can be used to mimic H3K56ac since it introduces a similar change in charge and increases nucleosome unwrapping similarly to H3K56ac^32^. We find that H3K56Q does not impact H1.0 binding to nucleosomes (Supplementary Fig. 8), and that H3K56Q prevents the influence of H1.0 on Gal4 binding within the nucleosome. These similar results on H3K56Q and H3K56ac suggest that the impact of these histone modifications on H1.0 regulation of nucleosome unwrapping is due to their increase in the probability that the nucleosome is partially unwrapped and not a specific interaction between H1.0 and the acetyllysine present within H3.

To confirm that H3K56ac does not influence H1.0 binding to nucleosomes, we used the Cy3-only labelled nucleosomes containing Cy3-L DNA and H3K56Q to detect H1.0 binding to linker DNA. As with unmodified nucleosomes, Gal4 titrations without H1.0 show that the Gal4 does not introduce an increase in PIFE with H3K56Q nucleosomes (Supplementary Fig. 8). We then carried out Gal4 titrations with 20 nM H1.0. The PIFE is increased due to H1.0 binding, but remains constant as Gal4 binds within the nucleosome (Supplementary Fig. 8). This shows that H1.0 remains bound to linker DNA of H3K56Q nucleosomes as Gal4 binds within these nucleosomes. Combined, our measurements suggest that both H3K56ac and H3K56Q abolish H1.0 regulation of DNA accessibility by preventing the H1.0-induced reduction in the probability that the nucleosome is partially unwrapped, which exposes the Gal4 site for Gal4 binding.

### H3K122ac does not alter H1.0 regulation of DNA accessibility

H3K122ac is positioned near the dyad symmetry axis of the nucleosome^3^, reduces nucleosome stability^30^,^33^ and is located within gene promoters of actively transcribed genes^36^. Since the WHD domain of H1.0 binds near the nucleosome dyad symmetry axis, we considered the possibility that H3K122ac could influence H1.0 regulation of Gal4 binding within the nucleosome. We prepared Cy3–Cy5 labelled nucleosomes with H3K122ac and Cy3-L DNA. We carried out H1.0 titrations with these nucleosomes and find that H3K122ac does not alter H1.0 binding to the nucleosomes (Fig. 5c, Table 1). We then carried out Gal4 titrations with nucleosomes containing H3K122ac and a range of H1.0 concentrations (0–20 nM). We find that H3K122ac does not reduce the impact of H1.0 on Gal4 binding (Fig. 5d,e, Table 1). Therefore, while H3K122ac reduces nucleosome stability and facilitates transcription, it does not directly regulate H1.0 function.

## Discussion

Employing a combination of FRET and PIFE ensemble experiments, we have investigated the influence of H1.0, H1.2 and H1.*x* on nucleosome unwrapping fluctuations. Specifically, we find that these H1 isoforms regulate nucleosome unwrapping without dissociating from the linker DNA, thus inhibiting but not completely blocking TF binding within partially unwrapped nucleosomes. Using synthetic and semisynthetic H3 histones with specific PTMs, we also determined that H3K56ac, an entry–exit region PTM, abolishes H1.0 regulation of TF binding within partially unwrapped nucleosomes but that acetylation at the nucleosome dyad does not. This suggests an interplay between different modes of regulation for TF binding in the nucleosome.

While many promoters are nucleosome free^17^, many regulated genes contain nucleosomes with TF-binding sites within the entry–exit region of the nucleosome^32^,^51^,^52^. The work here impacts our understanding of how gene promoters with TF-binding sites located within nucleosomes are regulated. These results point to two key consequences for linker histone regulation of chromatin accessibility of these genes.

**Figure 6:**
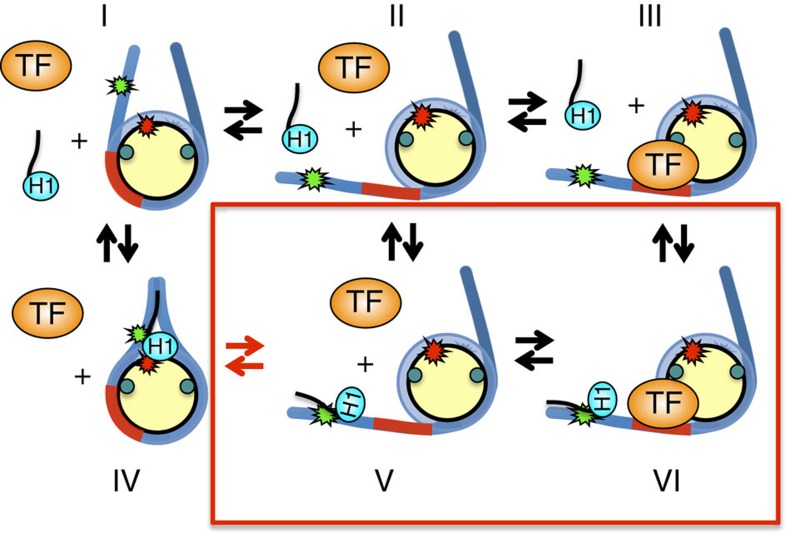
Model of H1 repression of TF binding to nucleosomes. Without H1 bound (state I), the nucleosome unwraps exposing the TF target site (state II) so the TF can bind (state III). H1 binding increases the amount of DNA wrapped into a nucleosome and blocks TF binding (state IV). However, if H1 releases from the nucleosome dyad region (state V), then the nucleosome can unwrap and permit TF binding (state VI). Our results indicate that states V and VI occur such that H1 does not dissociate from the linker DNA, while the nucleosome is partially unwrapped and bound by a TF. H1 reduces the probability of the nucleosome being in a partially unwrapped state, thereby suppressing TF occupancy. Furthermore, our results indicate the increase in nucleosome unwrapping probability by the acetylation of H3K56 (teal circles) counteracts the decrease in nucleosome unwrapping probability by H1 (red arrows), which eliminates H1 repression of TF binding.

The first consequence is that regulation by H1 isoforms do not require complete H1 dissociation. Figure 6 shows a diagram of how we propose H1 functions. In the top row, H1 is not bound (state I), allowing the nucleosome to thermally unwrap exposing the TF-binding site (state II), permitting the TF to bind (state III). In the bottom row, H1 remains bound to the linker region (state IV). If state IV were to block nucleosome unwrapping, then transition to state I would be required for TF binding. However, we find that the nucleosome partially unwraps with H1 bound to linker DNA (state V), allowing the TF to bind to its target sequence (state VI) while H1 remains bound. This observation of states V and VI demonstrates that chromatosomes undergo unwrapping fluctuations similarly to nucleosomes, albeit at a lower probability. This unwrapping provides proteins limited access to DNA sites within chromatosomes. Interestingly, H1 alters but does not block chromatin remodelling^53^,^54^,^55^, which suggests that H1 does not statically wrap nucleosomes. Instead, nucleosomes with H1 appear to remain dynamic so that they can still be remodeled, which is consistent with our finding that nucleosomes undergo unwrapping/rewrapping fluctuations while H1 is bound.

H1 and DNA interact with a dissociation constant of about 20 nM (refs 45, 46), which is similar to the H1 dissociation constant that we and others have measured with mononucleosomes and nucleosome arrays^45^,^46^,^56^. In contrast, the WHD of H1.0 interacts within the nucleosome dyad region with a much higher dissociation constant of about 1 μM (ref. 12). Given that the WHD of H1.0 binds with a much lower affinity than full-length H1.0, the WHD appears to transiently dissociate from the nucleosome dyad, while the C-terminal tail remains bound. Our observation that H1.0, H1.2 and H1.*x* remain bound to linker DNA, while Gal4 binds combined with these previous results suggest that the H1 C-terminal tail regulates the binding and dissociation of H1 through its interactions with the linker DNA, while the H1.0 WHD domain interactions near the nucleosome dyad influences nucleosome unwrapping and DNA accessibility.

Previous studies of H1 isoforms indicate that their relative affinities^25^ and exchange rates^26^ vary by over an order of magnitude. However, we find that H1.0, H1.2 and H1.*x* all bind nucleosomes with similar affinities. An important difference is that we studied recombinant H1 isoforms that do not contain PTMs, while these previous studies investigated histone isoforms within cells or extracted from cells, which will be post translationally modified^57^. This suggests that H1 PTMs could be important in regulating linker histone exchange and affinities. Furthermore, PTMs could enhance and/or reduce the twofold difference in TF occupancy we observe between H1.2 and H1.0/ H1.*x*. Future studies with linker histones containing well-defined PTMs will be required to determine their impact on linker histone function.

The second consequence for linker histone regulation of chromatin accessibility is that H3K56ac abolishes H1.0 regulation of nucleosome unwrapping. This suggests that the precise set of PTMs within the nucleosome can significantly impact H1.0 regulation and highlights the importance of studying nucleosomes with well-defined PTMs. Furthermore, since H3K56ac increases nucleosome unwrapping, our results suggest that changes in nucleosome unwrapping influence H1.0 function. Other factors that influence nucleosome unwrapping, including DNA sequence, histone variants and potentially other histone PTMs in the entry–exit region of the nucleosome may also influence H1.0 regulation of nucleosome unwrapping.

There are two general mechanisms by which histone PTMs function. PTMs can provide binding sites to recruit chromatin regulatory complexes^58^ such as chromatin remodelling factors and histone modifying enzymes^59^. Alternatively, PTMs can directly impact chromatin dynamics^60^ thereby regulating transcription factor occupancy^27^,^41^ and chromatin remodelling^61^. Our results highlight how direct changes in nucleosome dynamics can regulate a chromatin architectural protein. H3K56ac and perhaps other histone PTMs may therefore influence not only H1 but other chromatin architectural proteins such MeCP2 and the HMG proteins^62^,^63^,^64^. Finally, these findings provide a mechanism for how core histone post-translational modifications may define which regions of chromatin can be regulated by linker histones and perhaps other chromatin associated proteins.

## Methods

### Preparation of DNA molecules

Cy3-L, Cy3-R (Fig. 1a), Linker-L and Linker-R (Fig. 3a) DNA molecules for reconstituting nucleosomes for PIFE and FRET experiments were prepared by PCR with Cy3 labelled oligonucleotides from plasmid containing the Widom 601 nucleosome positioning sequence^42^ with the Gal4-2C-binding site (5′-CCGGAGGGCTGCCCTCCGG-3′)^65^ at bases 8–26 (Supplementary Table 1). Labelled oligonucleotides were prepared with Cy3 NHS ester (GE healthcare) at an amine-modified internal thymine and then HPLC purified on a 218TC C18 column (Grace/Vydac). Following PCR amplification, DNA constructs were purified by HPLC on a Gen-Pak Fax column (Waters).

### Preparation of core histones and histone octamer

Recombinant core histones H2A, H2A(K119C) and H2B were expressed in Rosetta (DE3) PlysS cells, while H3(C110A), H3(V35C, C110A), H3(K56Q,C110A), and H4 were expressed in BL21 (DE3) PLysS cells. The purification of each recombinant histone was done under denaturing conditions with gel filtration and ion exchange chromatography^66^. Plasmids containing Human H2A, H2B, H3 and H4 histones were generous gifts from Dr Karolin Luger (Colorado State University). Mutations into expression vectors to create H2A(K119C), H3(C110A), H3(V35C, C110A) and H3(K56Q, C110A) were introduced using a Quikchange Site Directed Mutagenesis Kit (Agilent Technologies).

Fully synthetic H3K56ac was prepared by sequential native chemical ligation of three synthetic peptides as previously described^31^,^67^, but with synthesis of peptide segments by Fmoc-SPPS chemistry on an alloc-protected diaminobenzoic acid linker to generate peptide thioesters^68^. After two ligation steps, H3K56ac was desulfurized, converting the introduced cysteines used in synthesis to native alanines. Protein purity and identity was assessed by RP-HPLC and MALDI-TOF MS.

H3K122ac histones were prepared by expressed protein ligation^33^,^67^. H3(1–109) was expressed as a fusion protein with the Mxe GyrA intein and the thioester generated by treatment with 2-mercaptoethanesulfonate. This H3 thioester was ligated with the H3(110–135) synthetic peptide segment bearing the acetyl modification at K122 (CAIHAKRVTIMPK(ac)DIQLARRIRGERA). H3(1–109) was co-purified with the full-length H3K122ac, but as described previously^33^, H3(1–109) cannot make essential contacts in the histone octamer and is eliminated during histone octamer refolding. H3K122ac purity and identity were assessed by SDS–PAGE and MALDI-TOF MS.

Each of the four histones were combined at equimolar ratios, refolded and purified by gel filtration chromatograph^66^. For refoldings using H3K122ac, equimolar ratios were calculated using the sum of H3(1–109) and H3K122ac protein content. H2A(K119C) and H3(V35C,C110A) containing histone octamer was labelled with Cy5-maleamide (GE-Healthcare)^31^. We obtained Cy5 labelling efficiencies of 85% for H2A(K119C) and 95% for H3(V35C,C110A). Briefly, lyophilized histones were unfolded in unfolding buffer (7 M Guandinium, 10 mM DTT) at a concentration of 5 mg ml^−1^ for 1–3 h, and then spun to remove aggregates. The absorption at 276 nm was measured for each unfolded histone to determine concentration. Histones were combined in a ratio of H2A:H2B:H3:H4 of 1.2:1.2:1:1 then unfolded by double dialysis into refolding buffer. The refolded octamer was then labelled with Cy5 maleamide as previously described^31^ before purification over a Superdex 200 (GE Healthcare) gel filtration column to remove excess dimer, tetramer and dye. The purity of each octamer was confirmed by SDS–PAGE, and labelling efficiency determined by Ultraviolet–visible.

### Preparation of linker histone H1 isoforms

The human histone H1.2 and H1.*x* ORFs (Invitrogen) were cloned into Gateway destination vector pDest-17 (Invitrogen) for 6 × His tagged recombinant protein expression. These vectors were then transformed into BL21 (DE3) pLysS *Escherichia coli* strains. For each H1 variant, 500 ml BL21 *E.coli* cultures were induced by L-arabinose, incubated at 37 °C for 3 h, then collected and lysed with denaturing lysis buffer (50 mM TrisCl, pH 7.5, 500 mM NaCl, 10% Glycerol, and 8 M urea). The supernatant was loaded onto a 1 ml HiTrap Chelating HP column (GE Healthcare) charged with Ni^2+^. Proteins bound to the column were washed with UreaMCAC-20buffer (50 mM TrisCl, pH 7.5, 500 mM NaCl, 10% Glycerol, 8 M urea, and 20 mM imidazole), then renatured by slowly passing a 5-ml linear gradient from 100% UreaMCAC-20 buffer to 100% MCAC-20 buffer (50 mM TrisCl, pH 7.5, 500 mM NaCl, 10% Glycerol, 0.5 mM PMSF and 20 mM imidazole) in 2 h. After renaturation, the column was washed with MCAC-50 buffer (20 mM TrisCl, pH 7.5, 500 mM NaCl, 10% Glycerol, 0.5 mM PMSF and 50 mM imidazole) and eluted with MCAC-500 buffer (20 mM TrisCl, pH 7.5, 500 mM NaCl, 10% Glycerol, 0.5 mM PMSF and 500 mM imidazole)^69^. The peak fractions collected from the nickel column were further purified using a Mono Q 5/50 GL column (GE Healthcare), eluted with a gradient from 100% low-salt buffer (50 mM TrisCl, pH 7.5, 50 mM NaCl, 10% Glycerol, and 0.5 mM PMSF) to 100% high-salt buffer (50 mM TrisCl, pH 7.5, 1 M NaCl, 10% Glycerol, and 0.5 mM PMSF) in 20 column volumes. The fractions containing purified 6 × His tagged H1.2 or H1.*x* were confirmed by Coomassie Brilliant Blue staining and western blotting with anti-His tag antibody (Pierce). Linker histone H1.0 was purchase from New England Biolabs.

### Preparation of Gal4

Gal4 protein was prepared according to published protocols^41^. The expression vector was prepared by cloning the gene for the first 147 residues of Gal4 (called Gal4(1–147) from the *Saccharomyces cerevisiae* genome into pET3a at the NdeI and BamH1 sites. Gal4(1–147) was expressed in Rosetta(DE3)pLysS *E. coli* cells (EMDMillipore) by inducing with 1 mM IPTG for 3 h. Cells were pelleted and resuspended in 50 ml per L of starting culture of Buffer A (50 mM Tris-HCl pH 8.0, 200 mM NaCl, 1 mM DTT, 10 μM ZnCl_2_, 1 mM phenylmethanesulfonyl fluoride (PMSF) with 20 μg ml^−1^ leupeptin, and 20 μg ml^−1^ pepstatin). The cells were lysed by sonication and centrifuged to remove cell debris. DNA was removed by precipitation with 0.35% v/v polyethyleneimine and then Gal4(1–147) was precipitated by 40% w/v ammonium sulfate. Gal4(1–147) was then resuspended in Buffer A and loaded onto a Sephacryl 200HR gel filtration column (GE Healthcare). Fractions containing Gal4(1–147) were dialysed against Buffer B (20 mM potassium phosphate pH 7.0, 10% v/v glycerol, 1 mM DTT, 10 μM ZnCl_2_ 1 mM PMSF) with 200 mM NaCl, and loaded directly onto a cellulose phosphate column equilibrated in Buffer B with 200 mM NaCl and eluted with a linear gradient of 200–800 mM NaCl. Fractions containing Gal4(1–147) were dialysed against Buffer C (25 mM Tris-HCl pH 7.5, 1 mM DTT, 10 μM ZnCl_2_ 1 mM PMSF) with 200 mM NaCl and then purified on a TSKgel SP5-PW (Tosoh Biosciences) column equilibrated with Buffer C with 200 mM NaCl and eluted with a linear gradient of 200–800 mM NaCl. Fractions containing Gal4(1–147) were dialysed against Buffer D (10 mM HEPES, pH 7.5, 200 mM NaCl, 10% v/v glycerol, 1 mM DTT, 10 μM ZnCl_2_, 1 mM PMSF) and stored at −80 °C.

### Preparation of nucleosomes

Nucleosomes were prepared by mixing histone octamer with DNA in a molar ratio of 0.8:1 octamer:DNA in 0.5x TE pH 8.0, 2 M NaCl, 1 mM Benzamidine hydrochloride in sample volume of 50 μl and reconstituted by salt double dialysis^66^. Dialysed nucleosomes were loaded onto a 5–30% w/v sucrose gradient and purified by ultracentrifugation on an Optima L-90K Ultracentrifuge (Beckman Coulter) with a SW41 rotor. Fractions containing correctly positioned nucleosomes were then collected and concentrated. Nucleosomes were then run on a 5% native acrylamide gel in 0.3 × TBE at 300 V for 1 h to verify purity (Fig. 1d).

### Electrophoretic mobility shift assay

H1 binding to nucleosomes was confirmed by EMSA 5 nM nucleosomes were incubated with 0–300 nM H1.0 (New England Biolabs) in 10 mM Tris-HCl pH 8.0, 130 mM NaCl, 10% Glycerol, 0.005% TWEEN20 in a 20 μl volume at 20 °C for 10 min. Samples were then resolved on a 4% polyacrylamide gel in 0.3 × TTE, 10% Glycerol.

### FRET measurements

TF binding to its target site within Cy–Cy5-labelled nucleosomes traps the nucleosome into a partially unwrapped state resulting in a reduction of FRET efficiency^27^,^31^. We used this decrease in signal to detect TF binding. We also used FRET to detect H1 binding since H1 binding induces an increase in the DNA wrapped into a nucleosome and a concomitant increase in FRET efficiency. We chose to use FRET measurements as the main tool for investigating H1 binding because this allows us to detect H1 binding in equilibrium and we can observe changes in the amount of DNA wrapped into the nucleosome. Fluorescence spectra were acquired with a Fluoromax4 (Horiba). H1 and Gal4 titrations were done with 5 nM nucleosomes in 10 mM Tris-HCl pH 8.0, 130 mM NaCl, 10% Glycerol and 0.005% TWEEN20. Samples were mixed in a 20 μl volume with 0–1,000 nM Gal4 and/or 0–300 nM H1 and allowed to incubate for 10 min at 20 °C before being measured. FRET efficiency measurements were determined by the (ratio)_A_ method^70^. Fluorescence emission spectra were measured as previously described^31^. We previously determined that nonspecific DNA binding of Gal4 does not reduce the FRET efficiency^41^ implying that our observed reduction in FRET is due to TF binding to its target sequence. We confirmed that H1.0 binding did not alter the Cy5 quantum yield and cause a change FRET efficiency by directly exciting Cy5 at 610 nm and observing that the Cy5 direct emission did not significantly change in the H1.0 titration (Supplementary Fig. 2).

We also measured the FRET efficiency at 0, 1, 5, 10 and 20 min following the addition of Gal4. This was done with 0 and 20 nM H1.0. We find that the FRET efficiency remains constant after 1 min (Supplementary Fig. 9). This indicates that Gal4 and H1.0 binding has reached equilibrium following a 10-min incubation.

Gal4 binds to DNA with a picomolar dissociation constant and to nucleosomes with sub nanomolar dissociation constant. Our fluorescence measurements require nucleosome concentrations of 5 nM. Therefore, Gal4 binds to nucleosomes stoichiometrically and we do not observe a change in the Gal4 *S*_1/2_ due to H3K56ac.

### PIFE measurements

PIFE measurements were done with nucleosomes prepared with Cy3 labelled DNA and unlabelled histone octamer. PIFE was used as a method of observing H1 binding that did not result in nucleosome wrapping. Fluorescence spectra were taken using an excitation of 510 nm. A reference spectrum of Cy3 labelled DNA only was fitted to each of the sample spectra to determine the relative fluorescence. H1 and Gal4 titrations were done with 5 nM nucleosomes in 10 mM Tris-HCl pH 8.0, 130 mM NaCl, 10% Glycerol and 0.005% TWEEN20. Samples were mixed in a 20 μl volume with 0–1,000 nM Gal4 and/or 0–300 nM H1 and allowed to incubate for 10 min at 20 °C before being measured.

## Additional information

**How to cite this article:** Bernier, M. *et al.* Linker histone H1 and H3K56 acetylation are antagonistic regulators of nucleosome dynamics. *Nat. Commun.* 6:10152 doi: 10.1038/ncomms10152 (2015).

## Supplementary Material

Supplementary Information Supplementary Figures 1-9 and Supplementary Table 1

## Figures and Tables

**Table 1:** Table of H1.0 and Gal4 *S*_1/2_ with unmodified and modified nucleosomes.

**Sample**	**H1.0** ***S***_**1/2**_ **(nM)**	**Gal4** ***S***_**1/2**_ **(nM) [H1.0]=0 nM**	**Gal4** ***S***_**1/2**_ **(nM) [H1.0]=3 nM**	**Gal4 S**_**1/2**_ **(nM) [H1.0]=10 nM**	**Gal4** ***S***_**1/2**_ **(nM) [H1.0]=20 nM**
Unmod	9±6	9.2±0.2	12.4±0.3	23±2	31±2
K56ac	14±7	6.5±0.4	9.9±0.7	12±2	10±2
K56Q	15±5	8.7±0.6	7.8±0.7	8±1	13±3
K122ac	14±7	10.5±0.5	14±2	23±2	50±10

For each experiment, data for three titrations were averaged and s.d. found. A Hill Curve was fit to the data using non-linear least squares fitting, and the *S*_1/2_ with error was extracted.

**Table 2:** Linker histone isoform *S*_1/2_ and their influence on Gal4 binding.

**Sample**	**H1** ***S***_**1/2**_ **(nM)**	**Gal4 S**_**1/2**_ **(nM) with [H1]=*****S***_**1/2**_
H1.0	9±6	23±2
H1.2	6±2	22±2
H1.*x*	13±4	48±2

For each experiment, data for three titrations were averaged and s.d. found. A Hill Curve was fit to the data using non-linear least squares fitting, and the *S*_1/2_ with error was extracted.
