# Supplementary material for: Linker histone H1 and H3K56 acetylation are antagonistic regulators of nucleosome dynamics
Source: Nat Commun. 2015 Dec 9;6:10152. doi: 10.1038/ncomms10152 (PMC4682114; doi:10.1038/ncomms10152)
Supplement: Supplementary Information — Supplementary Figures 1-9 and Supplementary Table 1 [file ncomms10152-s1.pdf]

Supplementary Figures

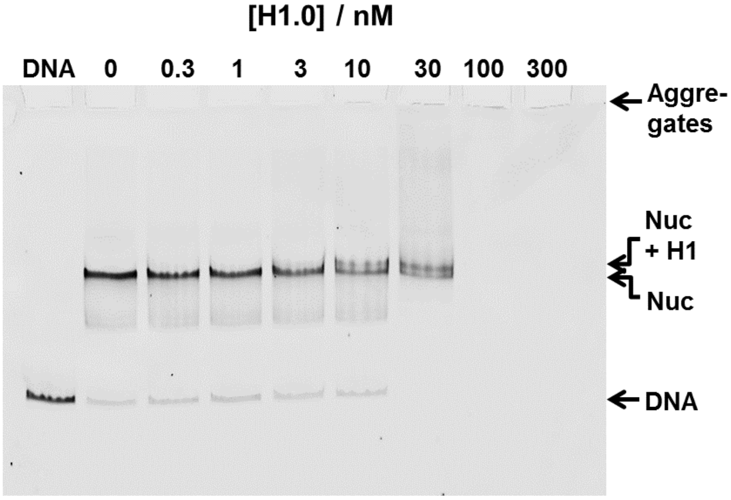

Supplementary Figure 1: H1.0 binding to unlabeled nucleosomes. 5 nM unlabeled nucleosomes were incubated with 0-300 nM H1.0 (New England Biolabs) in 10 mM Tris-HCl pH 8.0, 130 mM NaCl in a 20 μL volume at 20°C for 10 minutes. Samples were then mixed with a final concentration of 3% (v/v) ficoll before loaded onto a running 4% polyacrylamide gel in 0.3x TTE and resolved by running under constant voltage of 300V for 2 hours at 4°C. Each lane is labeled by the concentration of H1.0 in nM. The gel was stained using SYBR® Gold (Life Technologies) in 0.3x TTE and 2M NaCl before imaging by Typhoon scanner. The binding of H1.0 to unlabeled nucleosomes is similar to binding fluorophore labeled nucleosomes (**Fig. 2**).

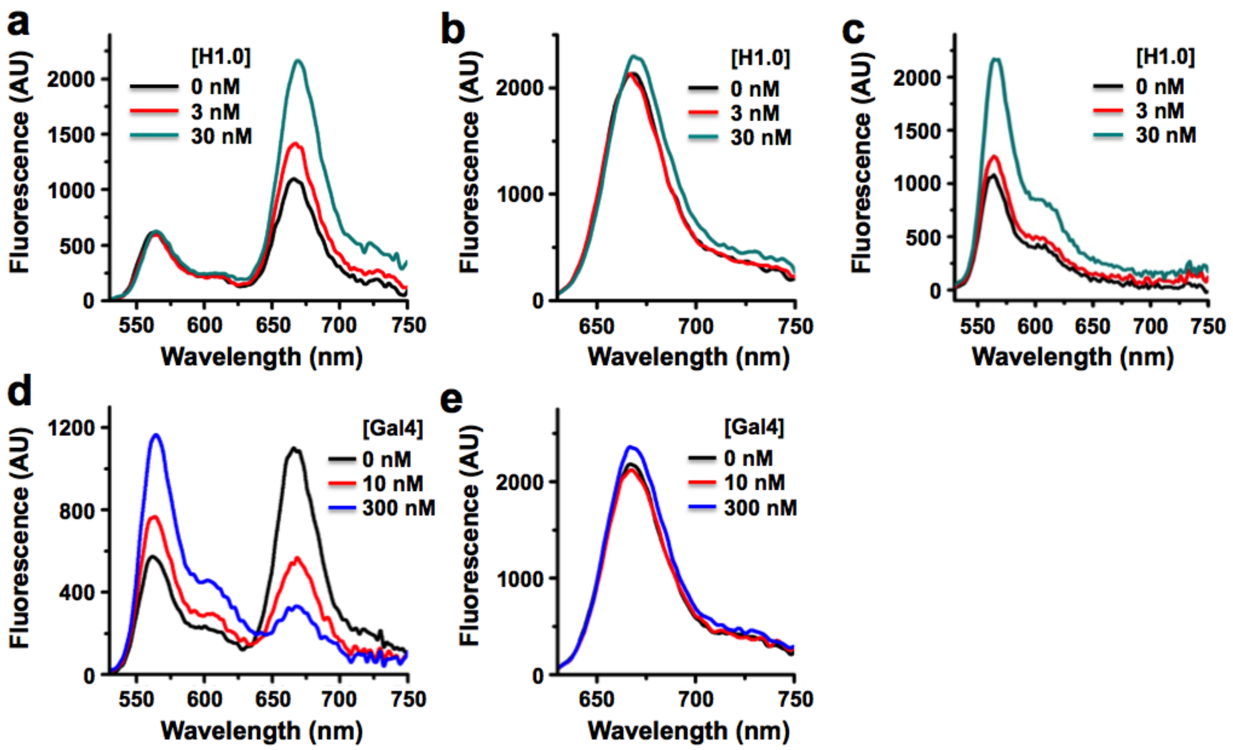

Supplementary Figure 2: Fluorescence spectra of FRET and PIFE nucleosomes. (**a & b**) Cy3-Cy5 labeled nucleosomes with 0 nM, 3 nM and 30 nM H1.0 excited at (**a**) 510 nm and (**b**) 610 nm. The peak at 660 nm in plot (**a**) shows increasing FRET efficiency as H1.0 concentration increases indicating increased wrapping as H1.0 binds. The constant emission spectrum of the 660 nm peak in plot (**b**) shows that H1.0 binding does not cause a change in FRET due to changes in the Cy5 quantum yield. (**c**) Cy3 only labeled nucleosomes with 0 nM, 3 nM and 30 nM H1.0. The Cy3 fluorescence increases with H1.0 concentration showing H1.0 interacting with the linker region. (**d & e**) Cy3-Cy5 labeled nucleosomes with 0 nM, 10 nM, 300 nM Gal4 excited at (**d**) 510 nm and (**e**) 610 nm. The peak at 660 nm in plot (**d**) shows increasing FRET efficiency as Gal4 concentration increases indicating decreased wrapping as Gal4 binds. The constant emission spectrum of the 660 nm peak in plot (**e**) shows that Gal4 binding does not cause a change in FRET due to changes in the Cy5 quantum yield.

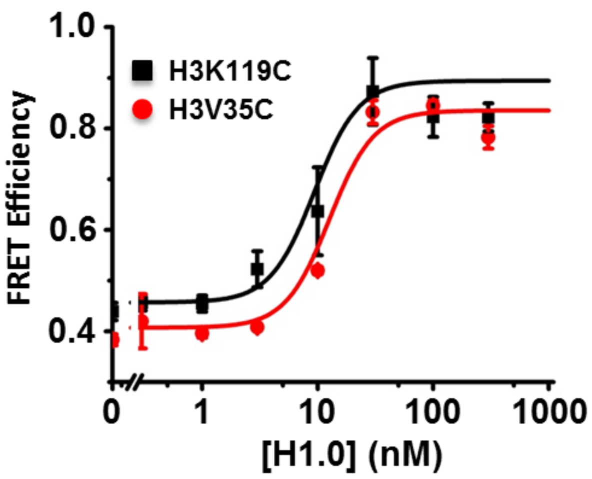

Supplementary Figure 3: H1 induced FRET change is due to increased nucleosome wrapping. H1 binding to nucleosomes with Cy5 at H3(V35C) compared to nucleosomes with Cy5 at H2A(K119C). The $S_{1/2}$ is not affected by Cy5 label location indicating that the FRET change is not caused by H2A C-terminal tail movement, but actually by nucleosome wrapping. The error bars were determined from the standard deviation of three independent measurements.

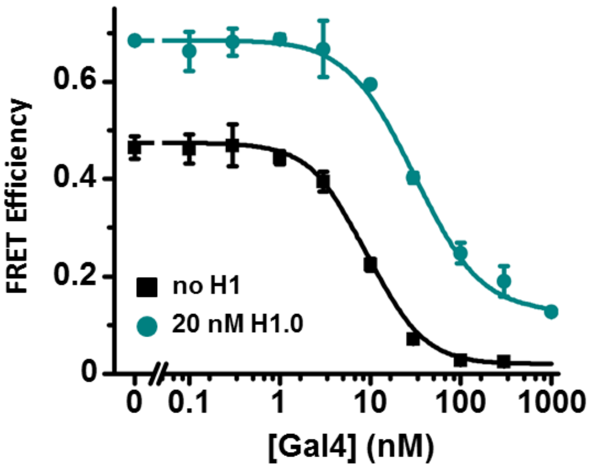

Supplementary Figure 4: H1 inhibits Gal4 binding to the site inside of a nucleosome. Absolute FRET Efficiency of Cy3-Cy5 labeled nucleosomes with Cy3-L DNA for increasing Gal4 concentrations with and without H1. The error bars were determined from the standard deviation of three independent measurements.

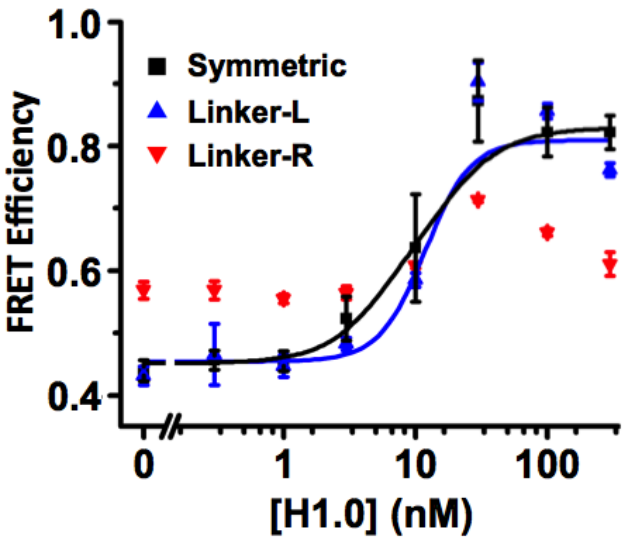

Supplementary Figure 5: H1 binding to asymmetric nucleosomes. FRET Efficiency of Cy3-Cy5 labeled nucleosome with asymmetric linker DNA as a function of H1.0 concentration. Linker-L nucleosome has linker DNA only on the same side as the Cy3 fluorophore. Linker-R nucleosome has linker DNA only on the opposite side of the Cy3 fluorophore. The FRET data were fit to Hill curves and the S1/2 for H1.0 binding is 12 ± 3 nM for the Linker-L nucleosome, and 11 ± 4 nM for the Linker-R nucleosome. The error bars were determined from the standard deviation of three independent measurements.

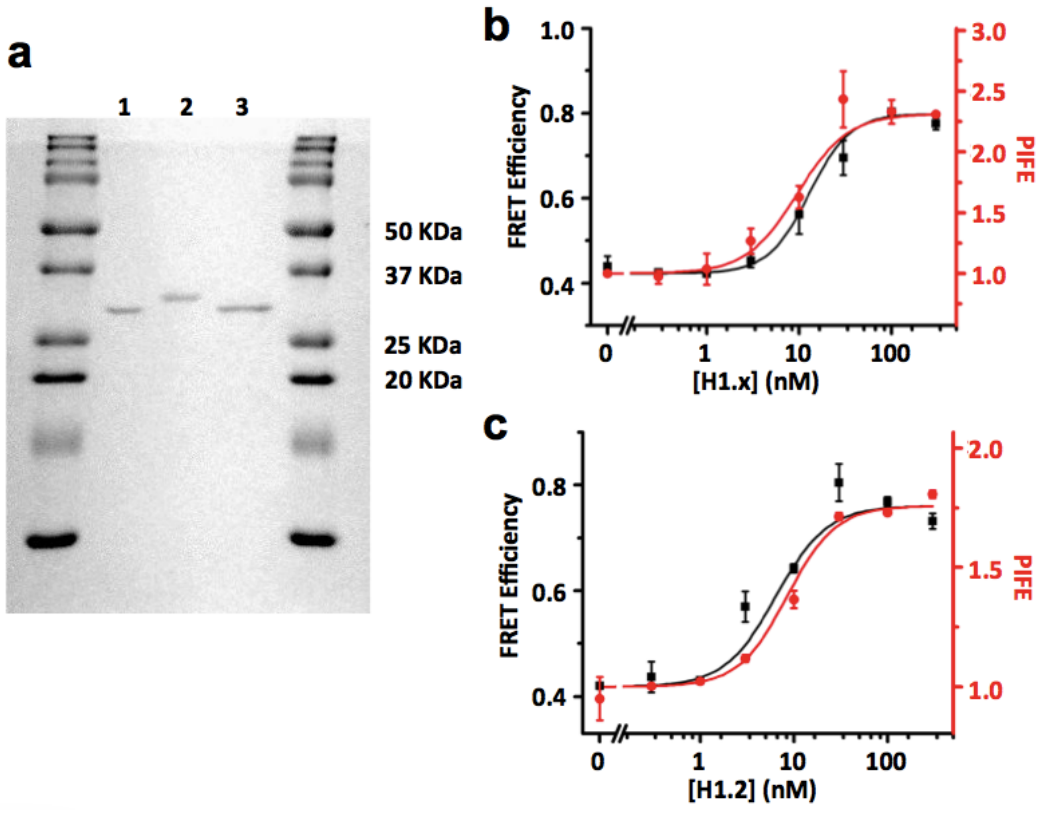

Supplementary Figure 6: Characterization of H1.2 and H1.x: (**a**) SDS-PAGE analysis of H1 proteins. Lane 1: H1.0 (NEB). Lane 2: H1.2. Lane 3: H1.x. The outside lanes contain the Bio-Rad Precision Plus Protein Standards (**b**, **c**) FRET Efficiency versus H1 concentration (black) measured with Cy3-Cy5 labeled unmodified nucleosomes containing Cy3-L DNA and PIFE signal versus H1 concentration (red) measured with Cy3 only unmodified nucleosomes with Cy3-L DNA for (**b**) H1.x and (**c**) H1.2. The error bars were determined from the standard deviation of three independent measurements.

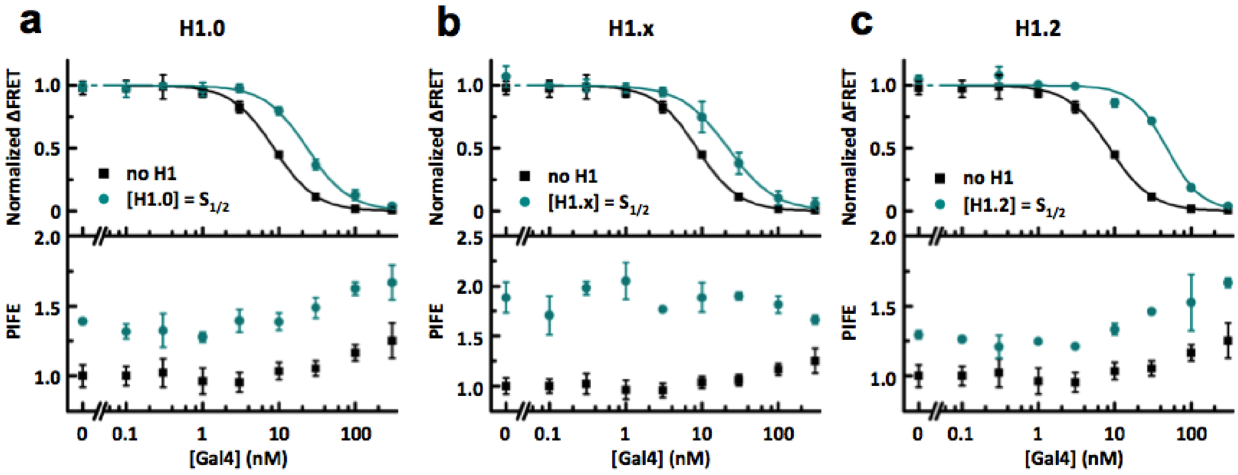

Supplementary Figure 7: FRET and PIFE measurements of Gal4 titrations with and without H1 isoforms. Relative FRET Efficiency of Cy3-Cy5 labeled unmodified nucleosomes (top panel) and relative fluorescence of Cy3 only unmodified nucleosomes (bottom panel) as a function of Gal4 concentration in the absences and presence of each histone isoform (**a**) H1.0, (**b**) H1.x and (**c**) H1.2. The concentration of each histone isoform was set equal to the measured $S_{1/2}$ for binding to nucleosomes (**Table 2**). The error bars were determined from the standard deviation of three independent measurements.

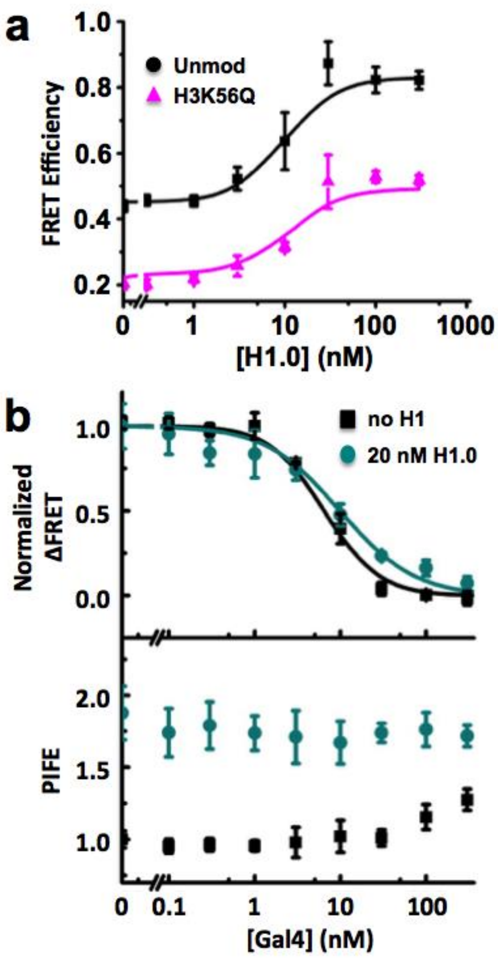

Supplementary Figure 8: PIFE data of Gal4 titrations with H3(K56Q) nucleosomes with and without H1. (**a**) FRET efficiency of Cy3-Cy5 labeled unmodified and H3(K56Q) nucleosomes as a function of H1 concentration showing increased nucleosome wrapping. (**b**) Relative FRET Efficiency of Cy3-Cy5 labeled H3(K56Q) nucleosomes as a function of Gal4 concentration in the presence and absence of H1. These data show no changes in the $S_{1/2}$ between 0 nM and 20 nM similar to H3(K56ac nucleosomes. (**c**) Relative Fluorescence of Cy3 only H3(K56Q) nucleosomes as a function of Gal4 concentration with and without H1. These PIFE data show that, like unmodified nucleosomes, H1 remains bound to the DNA as Gal4 binds within the nucleosome. The error bars were determined from the standard deviation of three independent measurements.

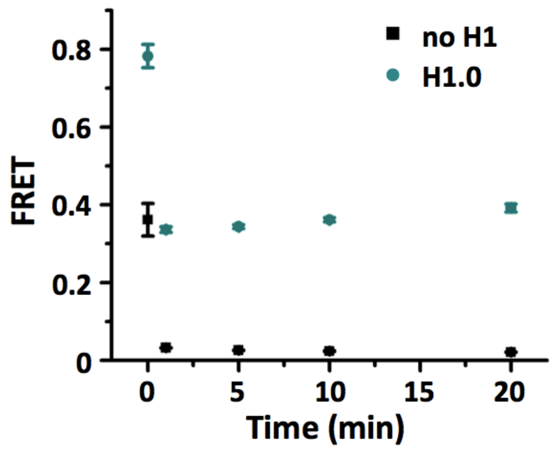

Supplementary Figure 9. FRET measurement with H1 and Gal4 with increasing time of incubation. 5 nM FRET nucleosomes were incubated with 100 nM Gal4 and 0 or 20nM H1.0 in a 60 μL volume for various period of time at 20°C before measuring FRET efficiency on the fluorometer. For time 0 data point, no Gal4 was added into the reaction mix, and the FRET efficiency was immediately measured after the nucleosomes were mixed with or without 20nM H1. The FRET efficiency is constant after 1 minute which indicates that the Gal4 and H1.0 binding reaches equilibrium during our incubation time of 10 minutes. The error bars were determined from the standard deviation of three independent measurements.

DNA	sequence
**Cy3-L**	5’-<br>AAGCTTGTCGACGAATTCAGATATAAGGAGGACACTGGGACA[AmC6dT]GCATCGG**CTGGAGACCGGAGGGCTGCCCTCCGGTCAATTGGTCGTAGACAGCTCTAGCACCGCTTAAACGCACGTACGCGCTGTCCCCCGCGTTTTAACCGCCAAGGGGATTACTCCCTAGTCTCCAGGCACGTGTCAGATATATACATCCTGT**ATAATGCATAGGGCAGTGAGTTGACGCTACAATCACGAATTCTGGATCCGATACGTAACGCGTCTGCAGCATGCG-3’
**Cy3-R**	5’-<br>AAGCTTGTCGACGAATTCAGATATAAGGAGGACACTGGGACATGCATCGG**CTGGAGACCGGAGGGCTGCCCTCCGGTCAATTGGTCGTAGACAGCTCTAGCACCGCTTAAACGCACGTACGCGCTGTCCCCCGCGTTTTAACCGCCAAGGGGATTACTCCCTAGTCTCCAGGCACGTGTCAGATATATACATCCTGT**ATAATGC**A**TAGGGCAGTGAGTTGACGCTACAATCACGAATTCTGGATCCGATACGTAACGCGTCTGCAGCATGCG-3’
**Linker-L**	5’-<br>AAGCTTGTCGACGAATTCAGATATAAGGAGGACACTGGGACA[AmC6dT]GCATCGG**CTGGAGACCGGAGGGCTGCCCTCCGGTCAATTGGTCGTAGACAGCTCTAGCACCGCTTAAACGCACGTACGCGCTGTCCCCCGCGTTTTAACCGCCAAGGGGATTACTCCCTAGTCTCCAGGCACGTGTCAGATATATACATCCTGT**-3’
**Linker-R**	AminoC6-5’-<br>**CTGGAGACCGGAGGGCTGCCCTCCGGTCAATTGGTCGTAGACAGCTCTAGCACCGCTTAAACGCACGTACGCGCTGTCCCCCGCGTTTTAACCGCCAAGGGGATTACTCCCTAGTCTCCAGGCACGTGTCAGATATATACATCCTGT**ATAATGCATAGGGCAGTGAGTTGACGCTACAATCACGAATTCTGGATCCGATACGTAACGCGTCTGCAGCATGCG-3’

Supplementary Table 1: DNA sequences. 601 sequence in bold. Gal4 binding site underlined. Amino group modification for Cy3 labeling highlighted.
